# Quantitative analysis of vitamin D and its main metabolites in human milk by supercritical fluid chromatography coupled to tandem mass spectrometry

**DOI:** 10.1007/s00216-019-02248-5

**Published:** 2019-12-12

**Authors:** J. M. Oberson, S. Bénet, K. Redeuil, E. Campos-Giménez

**Affiliations:** Nestlé Research, Route du Jorat 57, Vers-chez-les-Blanc, 1000 Lausanne, Switzerland

**Keywords:** Vitamin D, Human milk, Supercritical fluid chromatography, Tandem mass spectrometry

## Abstract

A novel method to quantitate vitamin D and its main metabolites (vitamin D3, vitamin D2, and their 25-hydroxy metabolites) in breast milk by supercritical fluid chromatography has been developed and fully validated. A small volume of sample (1 mL) is subjected to ethanolic protein precipitation and liquid-liquid extraction. Final extracts are derivatized with 4-phenyl-1,2,4-triazoline-3,5-dione and vitamin D derivatives analyzed by supercritical fluid chromatography hyphenated to tandem mass spectrometry with atmospheric pressure chemical ionization. Multiple reaction monitoring is used for quantitation. Separation conditions were optimized using a gradient of methanol-water-ammonium formate into carbon dioxide. Make-up solvent was methanol containing ammonium formate. The quantitation limit reached levels as low as 50 pmol/L, with intra- and inter-day relative standard deviations lower than 15% and 20% for all analytes. Accuracy was evaluated by spiking experiments and was well within acceptability ratios (± 15%). The method was then applied to a subset of commercially available human milk samples. The newly developed method provides opportunities to determine the nutritional status of mother-infant dyads from a non-invasive measure, or for interventional or observational studies building knowledge on the composition of human milk.

**Electronic supplementary material:**

The online version of this article (10.1007/s00216-019-02248-5) contains supplementary material, which is available to authorized users.

**Figure:**
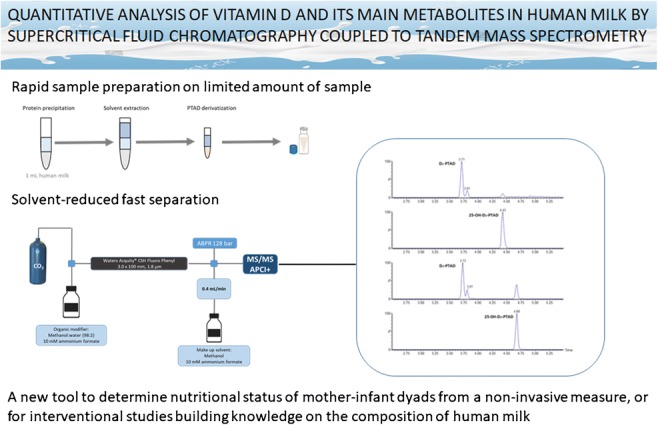
Graphical abstract

## Introduction

**Fig. 1:**
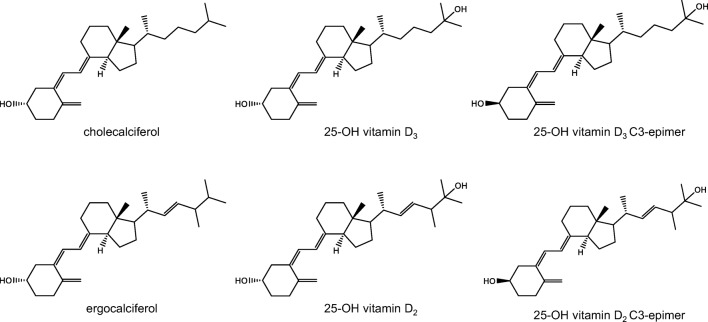
Chemical structure of vitamin D parent forms and main circulating metabolite 25-hydroxy vitamin D, together with C3 inactive epimer

Vitamin D is produced in humans from its precursor 7-dehydrocholesterol by the action of sunlight [1]. The photoconversion of the precursor yields previtamin D, which is converted to vitamin D by a temperature-dependent process. Vitamin D is transported to the liver and hydroxylated to 25-hydroxyvitamin D (25OHD), the main circulating form. In the kidney, 25OHD is metabolized into the active form 1,25-dihydroxyvitamin D. Two forms of vitamin D are present in nature: cholecalciferol (vitamin D3), produced in mammals, and ergocalciferol (vitamin D2), produced in plants, fungi, and yeast. Both forms differ structurally by only a double bond and a methyl group in the side chain (Fig. 1). The biological activity of both forms is considered equivalent in humans, although there is controversy around this statement [2–4]. International recommendations on food fortification (Codex Alimentarius CAC/GL 10/1979) do not distinguish between the two forms and thus, both need to be considered.

The first attempts to quantify vitamin D in human milk using chemical methods took place in the 1980's. Hollis *et al*. [5] used HPLC coupled to competitive protein binding detection and were first to quantitate vitamin D and its major metabolites. Several authors continued their work supporting the original idea that vitamin D and its 25-hydroxy derivatives constitute the main contributors to the vitamin D activity of human milk [6–9]. Nowadays, the most popular technique to analyze vitamin D in biological samples, besides immunoassays, is liquid chromatography coupled with tandem mass spectrometry [10–15]. Numerous methods have been published for human plasma or serum using liquid chromatography, but only a few for the analysis of milk [16–18], some of them [16, 17] using sample volumes larger than what is usually available from clinical studies (up to 10 mL).

Supercritical fluid chromatography (SFC), although originally reported more than 50 years ago [19], has only been used sporadically until the 1980's. With new developments on safer and more robust instrumentation in the later decade, it has become a more popular technique in the field of bioanalysis and it is slowly replacing liquid chromatography (LC). Modern SFC uses subcritical carbon dioxide as the main mobile phase, which is modified by a gradient of polar organic solvents. Carbon dioxide shows clear advantages over the previously used fluids, it is safe and cheap, and its supercritical state is easily reached (31.1 °C, 73.9 bar). When compared to LC, SFC separations are, in general, faster, provide sharper peaks, and improved resolution [20], meaning higher throughput and better efficiency [21, 22]. SFC hyphenated to tandem mass spectrometry (SFC-MS/MS) methods for the analysis of vitamin D and its metabolites in serum or plasma [23, 24] or vitamin D in fortified food products [25] have been published but, to the best of our knowledge, no methods applied to the analysis of human milk are available.

In the present work, we aimed at developing a novel and fully validated method to quantify vitamin D and its main metabolites by supercritical fluid chromatography using less than 1 mL of human milk.

## Experimental

### Chemicals

Solvents used in sample preparation (acetone for HPLC, ethanol for chromatography, ethyl acetate reagent grade, n-hexane for chromatography, isooctane for analysis) or for chromatographic analysis (acetonitrile and methanol for LC-MS), as well as reagents (formic acid *puriss.*, 4-phenyl-1,2,3-triazoline-3,5-dione (PTAD), ammonium formate for spectroscopy) were sourced from Sigma-Aldrich (Buchs, Switzerland). Pressurized CO_2_ (99.9%) was purchased from Carbagas (Gümligen, Switzerland).

### Human milk samples

Commercial milk samples from human donors were obtained from Lee Biosolutions (Maryland Heights, USA). Samples were received frozen (− 20 °C), thawn by placing them on a water bath at 40 °C for 1 h, mixed by stirring, aliquoted into 2-mL aliquots, and stored at − 80 °C prior to being used for development purposes. Maximum storage time was 3 months.

### Standards and calibration solutions

25-Hydroxy cholecalciferol (25OHD3) (50 μg/mL), 25-hydroxy ergocalciferol (25OHD2) (50 μg/mL), cholecalciferol (D3) (100 μg/mL), and ergocalciferol (D2) (1 mg/mL) were purchased as ready-to-use solutions from Sigma-Aldrich (Buchs, Switzerland). Commercial solutions were combined into a standard solution containing 1 ng/mL of each of the four derivatives in ethanol.

Isotope labelled internal standards (^2^H_6_-D3, ^2^H_6_-D2, 25OH^2^H_6_-D3, 25OH^2^H_6_-D2) were obtained from Chemaphor (Ottawa, Canada). Separate stock internal standard solutions (100 μg/mL in ethanol) were prepared and combined into an internal standard (IS) solution containing 20 ng/mL of ^2^H_6_-D3, ^2^H_6_-D2, 25OH^2^H_6_-D3, and 25OH^2^H_6_-D2. All solutions were kept at − 18 °C before use, for a maximum of 1 year.

Calibration standards were constructed by combining 25 μL of the IS solution and incremental volumes of the standard solution into 1.5-mL Eppendorf® tubes, drying over a stream of nitrogen and redissolving into 1 mL of isooctane for PTAD derivatization. Twenty-five microliters of PTAD solution (10 mg/mL in acetone) was added; the tubes were shaken and placed in the darkness for 1 h. After the addition of 100 μL of acetonitrile-water (80:20, v/v), the tubes were vortexed and centrifuged at 10,000×*g* for 5 min for phase separation. Final concentrations after derivatization (final volume 100 μL) were 0, 0.2, 0.5, 1, 2, 5, and 10 ng/mL.

Additional metabolites used for method development (1,25-dihydroxy-D2, 1,25-dihydroxy-D3, 24,25-dihydroxy-D2, and 24,25-dihydroxy-D3) were purchased from Sigma-Aldrich (Buchs, Switzerland). Individual solutions were prepared in ethanol, and PTAD derivatized following the same protocol as for the rest of standards. Final concentration after derivatization was 100 ng/mL.

### Choice of the chromatographic column

Nine chromatographic columns were chosen for screening. Individual derivatized solutions (100 ng/mL) were injected (3 μL) on all the columns. Methanol-water (98:2, v/v) containing 10 mM ammonium formate was used as organic modifier. Flow rate was adapted to each column tested according to the backpressure developed during the eluting gradient. The percentage of organic modifier was held at 0.5% from time 0 to 0.2 min and then slowly increased from 0.5% at 0.2 min to 50% in 8 min, maintained for 10 min at 50% and decreased to initial conditions for the next injection. Column temperature was maintained constant at 45 °C and atmospheric back pressure regulator (ABPR) at 128 bar. Make-up solvent was 10 mM ammonium formate in methanol at a flow rate of 0.4 mL/min. Once the chromatographic column was chosen, elution gradient was optimized to achieve best separation.

### Optimization of sample preparation

The effect of saponification (SN) as well as protein precipitation (PP) on the extraction efficiency was studied using the post-extraction addition method. IS solution (25 μL) was added to 1 mL of breast milk and subjected to either SN or PP followed by liquid-liquid extraction (LLE). In parallel, 1 mL of the same non-spiked milk was extracted and the IS solution (25 μL) added just before PTAD derivatization. LLE and SN conditions were based on previously published methodologies [26, 27] and adapted to the low volumes of sample available. Extraction solvent mixtures of slightly different polarities were also tested. Extraction efficiency was calculated from the relative difference between peak response in the extracts spiked before and after extraction.

### Sample preparation

Human milk samples were placed in a water bath at 40 °C for 1 h and thoroughly mixed prior to extraction.

Working into a Biosafety cabinet Class II, 1 mL of milk, 1 mL of ethanol, and 25 μL of IS solution were mixed in a 15-mL Falcon® polypropylene centrifuge tube. From this point, work was performed following common chemical safety regulations. A total of 2.5 mL of a mixture of n-hexane and ethyl acetate (90:10, v/v) was added; the tube was tightly closed and shaken in a Geno Grinder® for 3 min. Centrifugation at 2500×*g* for 5 min was applied for phase separation. The upper organic phase was then transferred into an 8-mL glass tube and dried down to almost dryness under a stream of nitrogen. The liquid/liquid extraction process was repeated, and the upper organic phases combined into the same glass tube. The combined organic phases were dried down to almost dryness under a stream of nitrogen, redissolved into 400 μL of the hexane-ethyl acetate mixture. The organic phase was transferred quantitatively into a 1.5-mL Eppendorf tube®. Two additional 400 μL portions of hexane-ethyl acetate mixture were added to rinse the glass tube and transferred into the same Eppendorf tube®. The combined portions were then dried under a stream of nitrogen. Hundred (100 ± 10) milligrams of sodium sulfate and 1 mL of isooctane were added. The tubes were vortexed and centrifuged at 10,000×*g* for 5 min. The dried liquid phase was transferred into another Eppendorf tube® for PTAD derivatization.

PTAD derivatization was adapted from a previously published protocol [25], and 25 μL of a PTAD solution (10 mg/mL in acetone) was added. The tubes were shaken and placed in the darkness for 1 h. One hundred microliters of acetonitrile:water (80:20, v/v) were added, and the tubes were vortexed for a few seconds and centrifuged at 10,000×*g* for 5 min. About 50 μL of the lower phase was transferred to a low-volume LC vial and 3 μL injected into the chromatographic system.

### Analytical instrumentation

Analysis was performed on an Acquity® UPC^2TM^ system (Waters, Milford, MA, USA) equipped with a binary pump, an autosampler, a column manager oven, an atmospheric back pressure regulator (ABPR), and a make-up pump coupled to a Waters Xevo^TM^ TQ-S mass spectrometer. The whole system was controlled by MassLynx^TM^ 4.1 software (Waters, Milford, MA, USA).

### Mass spectrometry instrumental conditions

Mass spectrometric detection was carried out on Atmospheric Pressure Chemical Ionization operating in positive mode at unit resolution (APCI^+^). Optimal parameters were needle corona at 4 kV, ion source temperature 150 °C, probe temperature 450 °C, cone gas flow 150 L/h, collision gas flow 0.15 mL/min. Argon was used as collision gas, while nitrogen was set as desolvation gas (650 L/h). Multiple reaction monitoring (MRM) was used and defined selected ions for detection and quantitation of each analyte are shown in Table 1.

**Table 1:** APCI+ parameters for each vitamin D-related compound. Precursor ion was the predominant molecular ion. The pair precursor ion/product ion was used for quantitation

	Cone voltage (V)	Collision energy (eV)	Precursor ion (*m/z*)	Product ion (*m/z*)
D2-PTAD	30	15	572.4	298.1
D3-PTAD	30	15	560.4	298.1
25OHD2-PTAD	30	17	570.4	298.1
25OHD3-PTAD	30	15	558.4	298.1
3*epi*-25OHD2-PTAD	30	20	570.4	298.1
3*epi*-25OHD3-PTAD	30	15	558.4	298.1
1,25diOHD2-PTAD	30	15	586.4	298.1
1,25diOHD3-PTAD	30	15	574.4	298.1
24,25diOHD2-PTAD	30	30	586.4	298.1
24,25diOHD3-PTAD	30	30	574.4	298.1

### Calculations

TargetLynx^TM^ software package (Waters, Milford, MA, USA) was used for automated peak integration and data processing. Isotope labelled internal standards were used for calibration on a solvent-based matrix. A weighed (1/x) linear regression model from peak area ratios (peak area of analyte/peak area of labelled internal standard) versus concentration was automatically constructed and used for calculations. Results were expressed as ng/100 mL according to final dilution factor (1 mL milk/100 μL).

### Method validation

The optimized method was submitted to validation following Guidance for Industry on Bioanalytical Method Validation (US Dpt. of Health and Human Services, FDA, May 2001). Selectivity, sensitivity, and calibration linearity were evaluated. Matrix effect was studied using the post-extraction addition method. A standard reference material (SRM 1849a Infant/Adult Formula) from the National Institute of Standards and Technology with certified vitamin D3 value (111 μg/kg ± 17 μg/kg) [28] was used as reference due to the absence of a human milk reference material. One gram of powder was diluted into a final volume of 1000 mL to provide a liquid sample containing a concentration of vitamin D3 close to the expected values in human milk (11.1 ng/100 mL). One milliliter of the diluted powder was analyzed on duplicate on ten different days. The accuracy and precision of the method were further evaluated by spiking a human milk sample at different concentration levels and carrying out the full sample treatment. Accuracy was evaluated as the difference between the amount in the spiked sample and the amount in the non-spiked sample *vs* the spiked concentration (recovery). Standard deviation of repeatability (Sr) and intermediate reproducibility (SiR) calculated according to ISO-5725 from the duplicate analysis were used as estimates of within- and between-day method variability (precision).

## Results and discussion

### Method optimization

#### Choice of metabolites and chromatographic conditions

Table 2 shows retention time of the PTAD derivatives on each of the columns screened (see Electronic Supplementary Material (ESM) Figs. S1 to S9 for example chromatograms). The choice of the metabolites to be included in the separation was based on previous reports. The main components of the vitamin D activity in human milk are the parent compounds (D2 and D3) and their 25OH metabolites [5–9], while the contribution of other metabolites (i.e., 1,25-dihydroxy-D2, 1,25-dihydroxy-D3, 24,25-dihydroxy-D2, and 24,25-dihydroxy-D3) is considered negligible [6, 7]. The reason to include them in the method development was to ensure that, even if they were present in very small quantities, they would not interfere with the main forms compromising method specificity. Methanol was the only organic modifier tested, since better separations and higher sensitivities have been reported using methanol as compared to acetonitrile in SFC [15, 17]. The introduction of methanol as organic modifier produced increased backpressure, but this effect was controlled by reducing total flow rate on the second part of the elution gradient. Based on previous experiences [25], ammonium formate and a small percentage of water were included in the mobile phase. Requirements for the choice of the column were mass and/or chromatographic separation. Special interest was given to metabolites that could potentially interfere with the most relevant compounds (D2, D3, 25OHD2, 25OHD3). This was the case of the separation between the inactive 3-*epi*-25OH-D (Fig. 1) and its biologically active isomer 25OH-D [29]. Some of the individual compounds were detected as double peaks (compounds with two retention times reported in Table 2), i.e., D2-PTAD on a Waters Torus^TM^ 1-AA column (ESM Fig. S1). PTAD derivatization produces two epimers, 6S and 6R, which are chromatographically separated depending on the stationary and mobile phases, as observed in Table 2. Although both peaks can be used for quantitation, their chromatographic separation might not be advantageous for quantitative purposes, as previously reported by other authors [15]. In order to maximize sensitivity, the columns providing single peaks for the main PTAD derivatives were preferred.

**Table 2:** Results of column screening. Injection of PTAD-derivatized individual solutions (100 ng/mL, 3 μL). Gradient elution, methanolwater (98:2) containing 10 mM ammonium formate into carbon dioxide. Retention times of each of the compounds in minutes, where two isomers of the PTAD derivative were separated the two values are shown. Column 1: Waters Torus^TM^ 1-AA, column 2: Waters BEH C18, column 3: Waters CSH^TM^ Fluoro Phenyl, column 4: Waters HSS C18 SB, column 5: Waters Torus^TM^ Diol, column 6: Waters BEH 2-EP, column 7: Nacalai Tesque cosmocore 2,6 cholester, column 8: Thermo Fischer Acclaim^TM^ C30, column 9: Chiral Technologies Chiralpak® AD-3

Column number		1	2	3	4	5	6	7	8	9
Particle size (μm)		1.8	1.7	1.7	1.8	1.7	1.7	2.6	3	3
ID (mm)		3	3	3	3	3	3	2.1	2.1	4.6
L (mm)		100	100	100	100	100	100	150	150	150
	Precurson ion (*m/z*)	Retention time (min)								
D2	572.4	3.60–3.71	3.02–3.04	2.54	3.18–3.30	2.82	3.74–3.77	6.44–6.68	2.76–2.89	5.30–5.80
D3	560.4	3.58–3.69	3.01–3.04	2.55	3.18–3.31	2.82	3.73–3.77	6.48–6.70	2.77–2.91	5.44–6.99
25OHD2	570.4	4.04–4.22	3.39	2.87	3.24–3.32	3.26	3.94–3.98	4.07–4.16	2.58–2.71	6.45
3*epi*25OHD2	570.4	4.18	3.36–3.39	2.81–2.91	3.25–3.32	3.25–3.28	3.96	4.38–4.44	2.61–2.76	6.80
25OHD3	558.4	4.06–4.26	3.41–3.43	2.94	3.27–3.35	3.32	3.96–4.02	4.07–4.16	2.61–2.75	6.48
3*epi*25OHD3	558.4	4.21	3.41–3.43	2.90–2.96	3.28–3.34	3.30–3.34	3.98–4.01	4.38–4.44	2.64–2.78	6.66
1,25diOHD2	586.4	4.47	3.70	3.11	3.32	3.55–3.60	4.17	5.60	2.91	8.00
24,25diD2	586.4	4.35–4.57	3.57–3.59	3.06	3.30–3.37	3.48	4.07–4.12	4.94–5.03	2.60–2.75	8.10
1,25diOHD3	574.4	4.51	3.70	3.15	3.32	3.59–3.63	4.20–4.27	5.60	2.93	8.40
24,25diOHD3	574.4	4.41–4.66	3.58–3.63	3.08–3.11	3.31–3.40	3.53	4.12–4.18	4.94–5.02	2.67–2.83	7.97–8.90

**Fig. 2:**
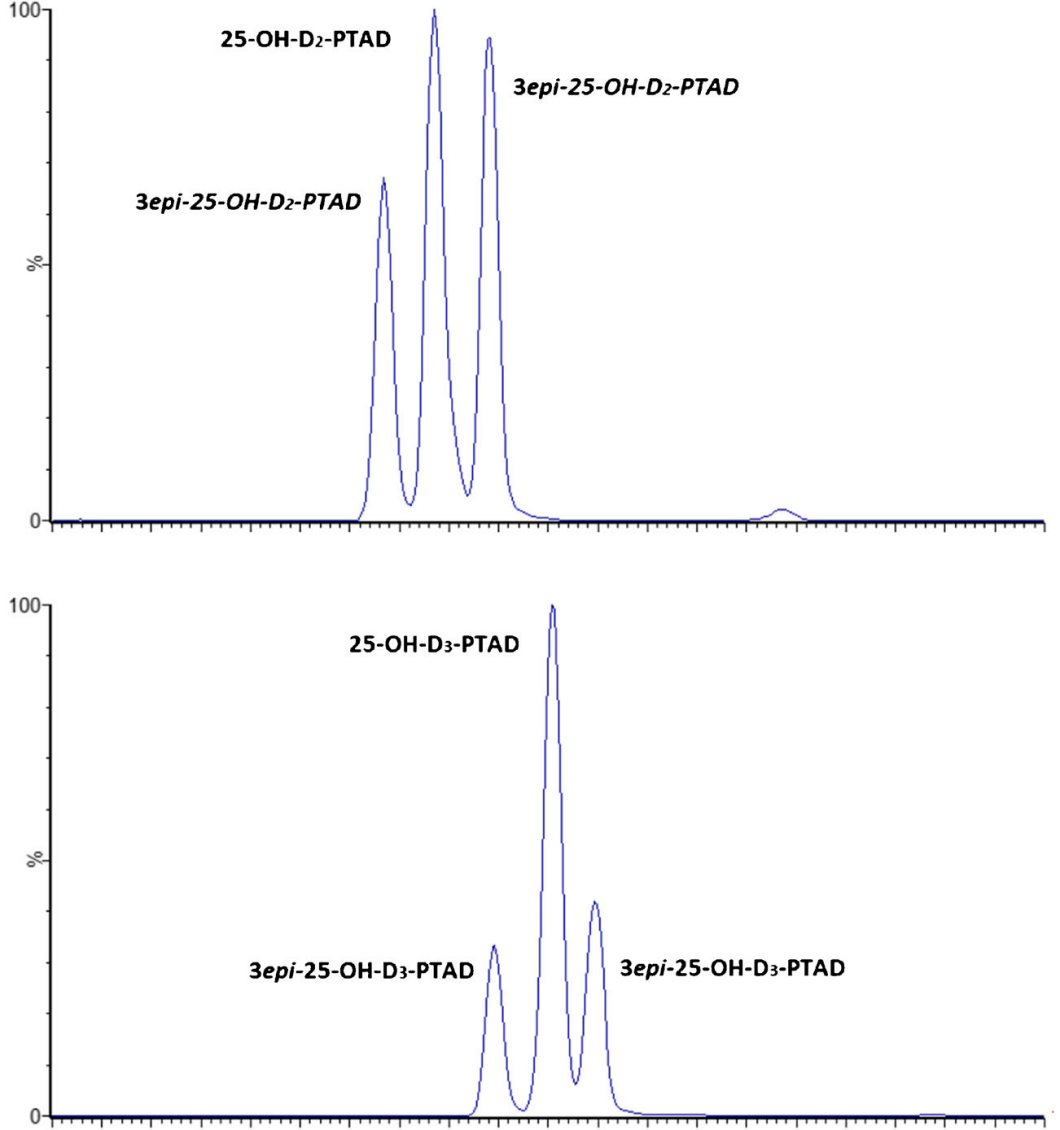
Chromatographic separation of 25-hydroxy vitamin D-PTAD derivatives and their C3 inactive epimer on a Waters CSH^TM^ Fluoro Phenyl column (1.7 μm, 3 × 100 mm). Gradient of methanol/water (98:2, v/v) containing 10 mM ammonium formate as organic modifier on carbon dioxyde. Column temperature 45 °C, Atmospheric Back Pressure Regulator 128 bar. Make-up solvent was 10 mM ammonium formate in methanol, flow rate 0.4 mL/min

Two columns (Chiralpak® AD-3 and CSH^TM^ Fluoro-Phenyl) met the initial requirements (mass and/or chromatographic separation of all compounds). The CSH^TM^ Fluoro-Phenyl column was retained for validation since it provided baseline resolution for all compounds not resolved by their mass fragmentation, with initially shorter elution times and better peak shapes than the Chiralpak® AD-3. Main metabolites (25OHD2 and 25OHD3) appeared as a single peak, facilitating integration and quantitation. After further optimization of the gradient, the 3-*epi* isomers of 25OHD were well separated, appearing as double peaks as seen in Fig. 2.

Validation was then carried out on a Waters Acquity® UPC^2TM^ CSH^TM^ Fluoro-Phenyl, 3.0 × 100 mm, 1.7 μm column. Compounds were eluted using an optimized gradient of methanol-water (98:2) containing 10 mM ammonium formate in CO_2_ (Table 3). Column temperature was set at 45 °C and ABPR at 128 bar. Total run time was 9 min. Make-up solvent was 10 mM ammonium formate in methanol at a flow rate of 0.4 mL/min.

**Table 3:** SFC optimized elution gradient. Mobile phase A: carbon dioxide. Mobile phase B: 10 mM ammonium formate in methanol:water (98:2)

Time (min)	Flow rate (mL/min)	Mobile phase A (%)	Mobile phase B (%)
0.0	3.00	99.5	0.5
0.5	3.00	99.5	0.5
5.0	2.75	92.0	8.0
6.0	1.75	70.0	30.0
7.0	1.75	70.0	30.0
7.8	1.75	99.5	0.5
8.5	3.00	99.5	0.5
9.0	3.00	99.5	0.5

#### Injection volume

**Fig. 3:**
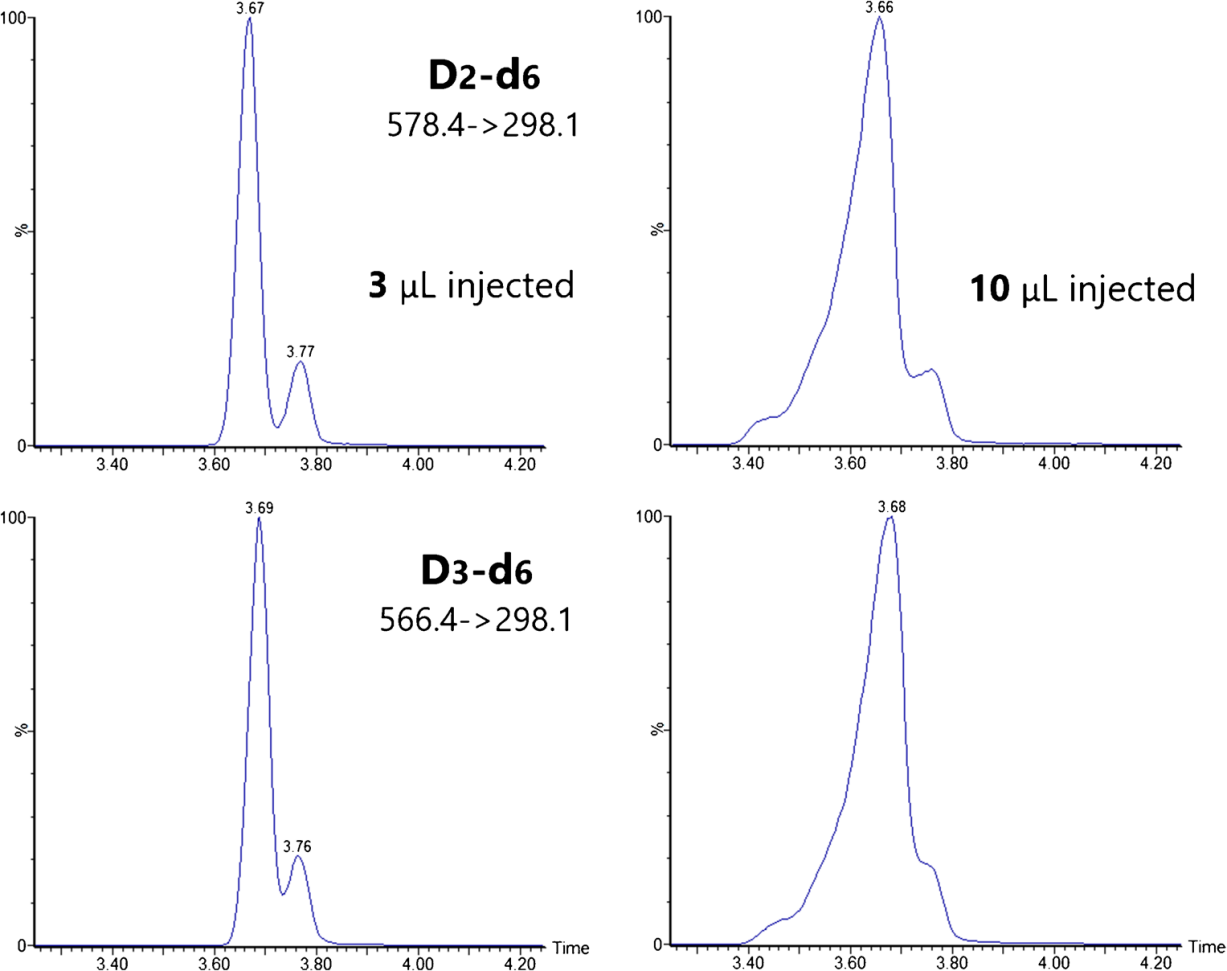
Effect of injection volume on peak shape of vitamin D2 and vitamin D3 on a Waters Acquity® UPC2TM CSHTM Fluoro-Phenyl, 3.0 × 100 mm, 1.7 μm column. Gradient elution with methanol containing ammonium formate as organic modifier

One of the drawbacks of SFC as compared to LC is the low volume of sample that can be injected without observing peak distortion [23]. This becomes critical when analyzing molecules present at low levels, in which increasing injection volume might be a valid option to maximize response. It has been shown that the volume that can be injected depends on the analyte, the organic modifier, and the chromatographic conditions and should be investigated on each method development [23, 30]. In our previous work [25], we demonstrated that PTAD derivatives of vitamin D did not show peak distortion at volumes as high as 10 μL on a Waters Acquity® UPC^2TM^ Torus^TM^ 1-Aminoanthracene, 3.0 × 100 mm, 1.8 μm column using same mobile phase as in the method here described. In an effort to explore all possibilities to increase sensitivity without compromising method performance, 3 and 10 μL of calibration standards were injected on the CSH^TM^ Fluoro Phenyl column. On the contrary to our previous observations on a Torus^TM^ 1-Aminoanthracene column with the same organic modifier and similar gradient, the injection volume could not be increased without peak distortion (Fig. 3), confirming the method dependency and the need for optimization on each individual development.

#### Sample preparation

Gomes et al. [17] reported the comparison between saponification (SN) and protein precipitation (PP) followed by liquid-liquid extraction (LLE) and showed better efficiency of the extraction when saponification was avoided, most likely due to degradation in alkaline media. Due to the difficulties to find samples completely devoid of vitamin D metabolites suitable to be used on spiking experiments, we tested the effect of SN as well as PP both followed by LLE, on the recovery of internal standards (Table 4). SN is the recognized method of choice for the analysis of the parent compounds (vitamin D) in milk products [31]. However, the recovery of the 25OH metabolites was strongly affected by the SN, as well as by the polarity of the extracting solvent. As expected, increasing the polarity of the extraction solvent increased the recovery, but the values did not exceed 70%. When SN was omitted, the results of the PP followed by LLE showed recovery rates between 70 and 107% for all metabolites and conditions tested, with slightly higher percentages when ethanol was used as protein precipitant rather than methanol. Ethanol was then preferred over methanol due to lower toxicity. Furthermore, the use of methanol produced, in some cases, emulsions during LLE, making phase separation more difficult than with ethanol. Increasing the polarity of the extraction solvent did not increase recovery on any of the analytes. Experiments on the effect of repeated extraction showed that two LLE steps were sufficient and a third cycle did not improve results. PP followed by LLE was then preferred over SN.

**Table 4:** Optimization of sample preparation using post-extraction addition method. Extraction efficiency calculated from the relative difference between peak response in a human milk sample spiked before and after extraction. Composition of extraction solvents and number of extraction cycles given in brackets. *PP* protein precipitation, *LLE* liquid-liquid extraction, *SN* saponification

	Extraction efficiency (%)			
	D3-d6	D2-d6	25-OHD3-d6	25-OH D2-d6
SN (ethanol), LLE (hexane)	67	73	10	5
SN (ethanol), LLE (hexane-ethyl acetate 9:1)	113	114	37	31
SN (ethanol), LLE (hexane-ethyl acetate 7:3)	144	136	65	68
PP (ethanol), LLE (hexane-ethyl acetate 9:1, 9:1)	107	112	106	101
PP (ethanol), LLE (hexane-ethyl acetate 9:1, 9:1, 9:1)	103	105	106	101
PP (ethanol), LLE (hexane-ethyl acetate 9:1, 4:1, 4:1)	67	71	85	96
PP (ethanol), LLE (hexane-ethyl acetate 9:1, 4:1, 3:1)	77	71	85	102
PP (methanol), LLE (hexane-ethyl acetate 9:1, 4:1, 3:1)	70	68	95	93
PP (methanol), LLE (hexane-ethyl acetate 9:1, 4:1, 3:1)	67	68	90	89

### Method performance

#### Selectivity

**Fig. 4:**
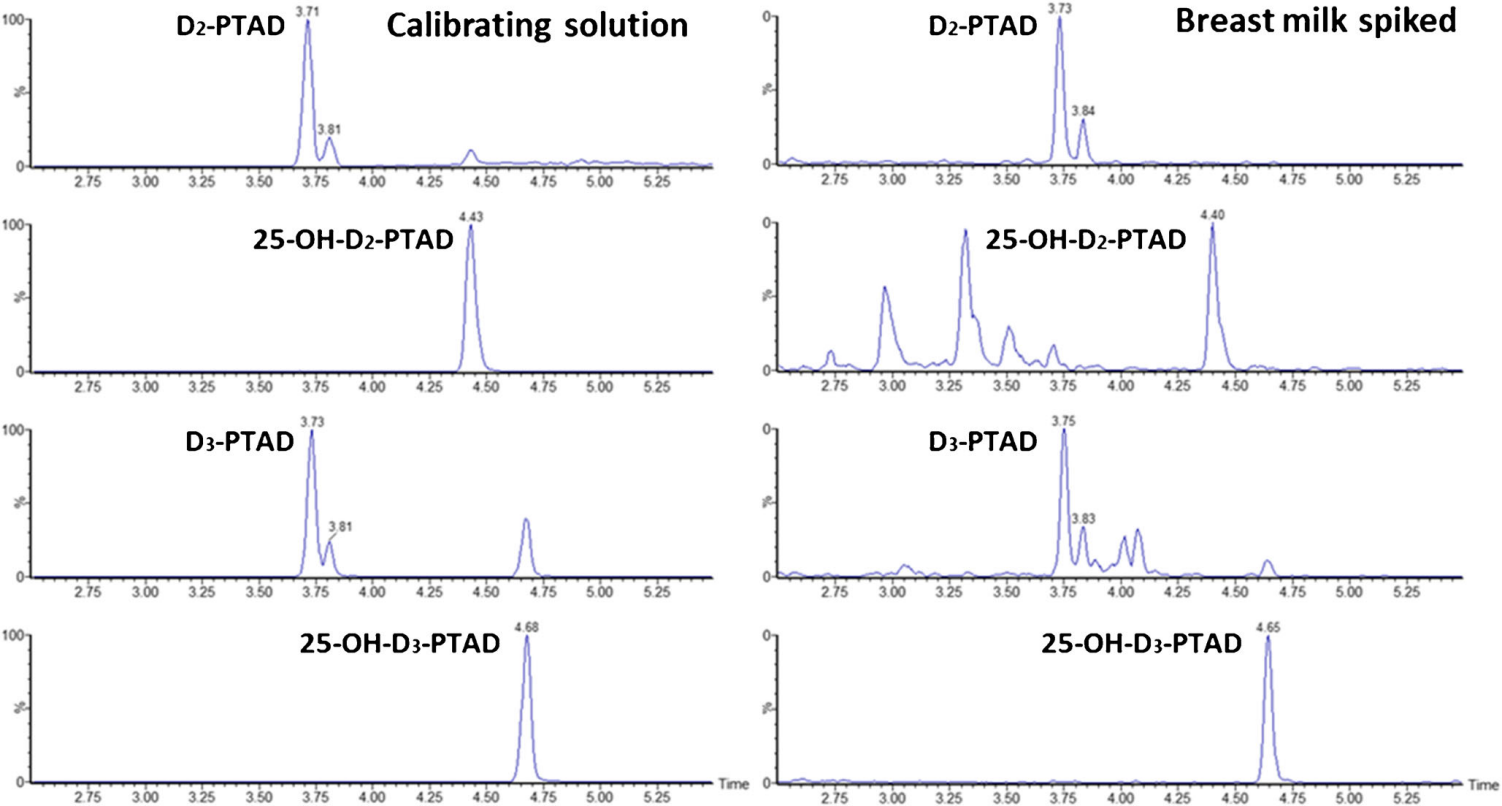
MRM chromatograms of a calibrating solution (2 ng/mL) and a spiked human milk (20 ng/100 mL)

Selectivity was ensured by the use of derivatization combined with chromatographic separation and tandem mass spectrometry. Indeed, PTAD derivatization efficiently removes interferences by shifting *m/z* to higher values and boosting sensitivity by increasing ionization efficiency [15, 25]. MRM chromatograms of each of the analytes showed absence of interfering peaks (Fig. 4). Due to the low concentrations, only one MRM transition, the predominant ion, could be monitored. The use of a second transition for confirmation could not be implemented, making separation from interferences critical for proper quantitation.

### Matrix effect and calibration

Since human milk composition may vary greatly, it evolves within lactation and shows extreme compositional differences between individuals; matrix effect can drastically change from one sample to the next. In the sample studied, matrix effect (ion suppression), evaluated using the post-extraction addition method, was lower than 20% for the parent compounds (D2 and D3) and lower than 5% for the metabolites (25OHD). Strategies to account for matrix effects include the use of matrix-match calibration. In an ideal situation, the exact same sample is used to build the calibration model. In routine work, a “pooled” sample representing the whole population is used to build the calibration. A calibration curve specific to each sample would be necessary to ensure accuracy. Due to the difficulties to obtain high volumes of milk to prepare calibration curves in addition to the sample itself, together with the burden of such practice in routine laboratories, the use of isotope labelled internal standards provides an excellent solution [32]. The commercial availability of isotope labelled internal standards for each of the compounds considered allowed us to perform calibration “in-solvent” instead of matrix-matched without compromising method performance.

### Linearity and limit of quantitation

Linearity of calibration (peak area ratio *vs* concentration) was demonstrated over the full analytical range (0.6 to 30 ng injected). Coefficient of determination (*r*^2^) was higher than 0.990 for all the analytes. Lower limit of quantitation (LLOQ) was established as the lowest level in the calibration curve (0.6 ng injected), which presented response 5 times higher than the instrumental noise together with accuracy (estimated *vs* nominal concentration) between 80 and 120%. All other levels of the calibration curve presented maximum 10% deviation from nominal concentration. Method lower limits of quantitation were established taking into account sample dilution factors (1 mL/100 μL) applied to the instrumental LLOQ and were as low as 2 ng/100 mL (equivalent to 50 pmol/L) for all analytes.

### Accuracy

The absence of certified reference materials undermines the determination of accuracy on the analysis of human milk. Standard Reference Material 1849a (a milk-based, hybrid infant/adult nutritional formula) from the National Institute for Standards and Traceability (NIST) provided a partial solution for at least one of the analytes (D3). Analysis of SRM 1849a diluted to vitamin D3 levels close to expected levels in human milk provided an average result of 9.42 ± 1.96 ng/100 mL (average of *n* = 10 duplicate determinations ± standard deviation, expressed on the diluted sample). This result is well within the certified limits 11.1 ± 1.7 ng/100 mL (calculated taking into account dilution). Despite the differences in matrix and the fact that vitamin D3 is fortified in the SRM, while it is naturally present in human milk, the results provided confidence on the approach. To our knowledge, no certified or reference human milk material containing 25OHD is commercially available.

Method accuracy was further evaluated from the analysis of QC samples at different levels on repeated non-consecutive days (Table 5). Method accuracy estimated from spiking at two different levels was within acceptability limits (± 15%) and not statistically different from 100% for all compounds and levels. Within and between-day variability was calculated (Table 5). Relative standard deviation of repeatability (RSr) was below 10%, except for the native 25OH derivatives concentration. In the case of 25OHD3 (19.4 ng/100 mL), it remained below 15%. 25OHD2 was present at LLOQ levels (2.7 ng/100 mL), showing variability as high as 37%.

**Table 5:** Intra- and inter-day variability on a human milk sample. Analysis performed on *n* non-consecutive days in duplicate. *n.a.* not applicable, *RSr* relative standard deviation of repeatability (within-day variability), *RSiR* relative standard deviation of intermediate reproducibility (between-day variability)

		Vitamin D2	Vitamin D3	25-OHD2	25-OHD3
Native amount	Average (ng/100 mL)	2.8	22.3	< 2	19.4
*n* = 6	RSr	37.3 %	10.8 %	n.a.	12.3 %
	RSiR	32.7 %	16.7 %	n.a.	13.7 %
Spiked (+ 20)	Average (ng/100 mL)	25.5	49.8	20.8	39.6
*n* = 10	Accuracy	112.0 %	117.7 %	104.2 %	100.4 %
	RSr	10.8 %	7.1 %	9.1 %	6.9 %
	RSiR	26.9 %	11.3 %	14.7 %	10.7 %
Spiked (+ 40)	Average (ng/100 mL)	44.7	n.a.	43.4	n.a.
*n* = 10	Accuracy	104.3 %	n.a.	108.5 %	n.a.
	RSr	9.6 %	n.a.	8.5 %	n.a.
	RSiR	14.9 %	n.a.	14.4 %	n.a.

### Application of the method to human milk samples

The method was applied to commercial human milk samples from healthy single donors (Lee Biosolutions, Maryland Heights, USA). The results (Table 6) show the presence of metabolites of vitamin D3 as the main contributors to the total vitamin D activity, while the presence of low levels of vitamin D2 occurs most likely from dietary intake of foods such as mushroom or from supplementation. The concentrations of the individual metabolites in milk correspond to maternal circulating levels and are expected to be around a hundred times lower than those [33, 34]. In the subset of samples here analyzed, all the results were consistent with previously reported data in Western populations [18]. It has been reported that minimal day-to-day variation is expected on the 25OH metabolites, while the amount of the parent compound may vary greatly with UV exposure or intake [34]. In this subset of samples, we confirmed this observation among different individuals, with a similar concentration of 25OHD3 in all the samples, consistent with expected levels, and very variable values on the parent forms.

**Table 6:** Vitamin D metabolites in a subset of commercial human milk samples. Antirachitic activity (ARA) calculated as 1 IU/L = 25 pg/mL vitamin D = 5 pg/mL vitamin 25OHD [18]. Average of triplicate analysis ± standard deviation

	D2	D3	25OHD2	25OHD3	ARA	% 25OH ARA
	ng/100 mL				IU/L	
Sample 1	4.2 ± 0.6	25.8 ± 0.8	< 2	18.7 ± 0.3	47.7	78%
Sample 2	3.8 ± 0.3	6.5 ± 0.8	< 2	18.7 ± 1.9	40.0	93%
Sample 3	4.0 ± 0.8	19.5 ± 0.9	< 2	12.8 ± 1.3	33.4	77%
Sample 4	3.7 ± 1.0	15.2 ± 1.7	< 2	25.7 ± 1.1	57.5	89%
Sample 5	6.5 ± 0.4	301.1 ± 27.3	< 2	25.9 ± 1.1	172.3	30%
Sample 6	3.5 ± 0.8	11.3 ± 0.9	< 2	14.8 ± 0.9	34.1	87%
Sample 7	3.5 ± 0.3	19.1 ± 1.8	< 2	23.5 ± 1.9	54.6	86%

## Conclusions

We present a novel SFC-MS/MS method for the quantitation of vitamins D2, D3, and their main metabolites 25OHD3 and 25OHD2 in 1 mL of human milk. All analytes were separated on a Waters CSH^TM^ Fluoro Phenyl column using a gradient of methanol-ammonium formate on carbon dioxide. Sample preparation ensured recovery of vitamin D metabolites and efficient removal of interfering compounds to achieve low detection limits. Quantitation limits were as low as 2 ng/100 mL of milk (corresponding to 50 pmol/L), with method accuracy demonstrated by recovery from spike experiments well within acceptability limits (100% ± 15%) at two concentration levels and complemented with the analysis of a standard reference material. Within- and between-day variability (represented by standard deviation of repeatability and reproducibility) were below 15% and 20% respectively.

The method was successfully applied to human milk samples showing good potential for application to large clinical studies. As many as 50 samples can be analyzed on a single run, automation of sample preparation would increase throughput but the possibilities were not explored at this time. The newly developed method provides opportunities to determine nutritional status of mother-infant dyads from a non-invasive measure, or for interventional or observational studies building knowledge on the composition of human milk.

## Electronic supplementary material

ESM 1 (PDF 476 kb)
