# Supplementary material for: Quantitative analysis of vitamin D and its main metabolites in human milk by supercritical fluid chromatography coupled to tandem mass spectrometry
Source: Anal Bioanal Chem. 2019 Dec 12;412(2):365–75. doi: 10.1007/s00216-019-02248-5 (PMC6992569; doi:10.1007/s00216-019-02248-5)
Supplement: Supplementary file 1 — (PDF 476 kb) [file 216_2019_2248_MOESM1_ESM.pdf]

Analytical and Bioanalytical Chemistry

Electronic Supplementary Material

Quantitative analysis of vitamin D and its main metabolites in human milk by supercritical fluid chromatography coupled to tandem mass spectrometry

J. M. Oberson, S. Bénet, K. Redeuil, E. Campos Giménez

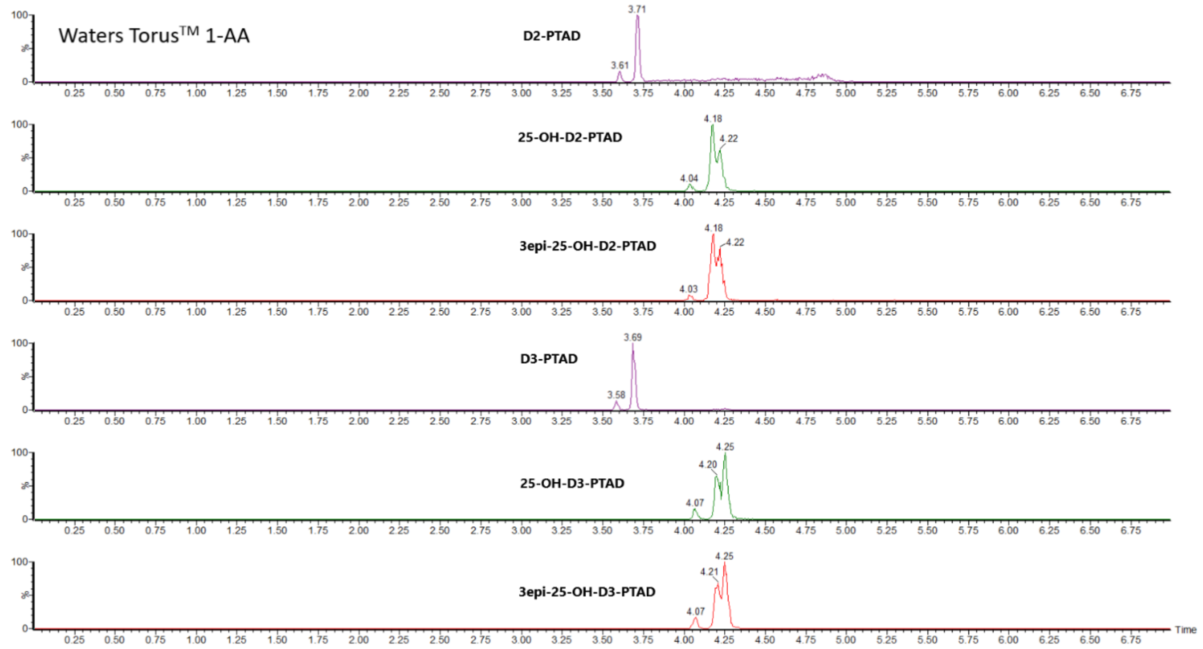

Fig. S1 Chromatogram obtained during column screening on column 1: Waters Torus^{TM} 1-AA, 3 mm x 100 mm, 1.8 µm. Injection of PTAD-derivatized individual solutions (100 ng/mL, 3 µL). Gradient elution, methanol:water (98:2) containing 10 mM ammonium formate into carbon dioxide. Retention time in minutes

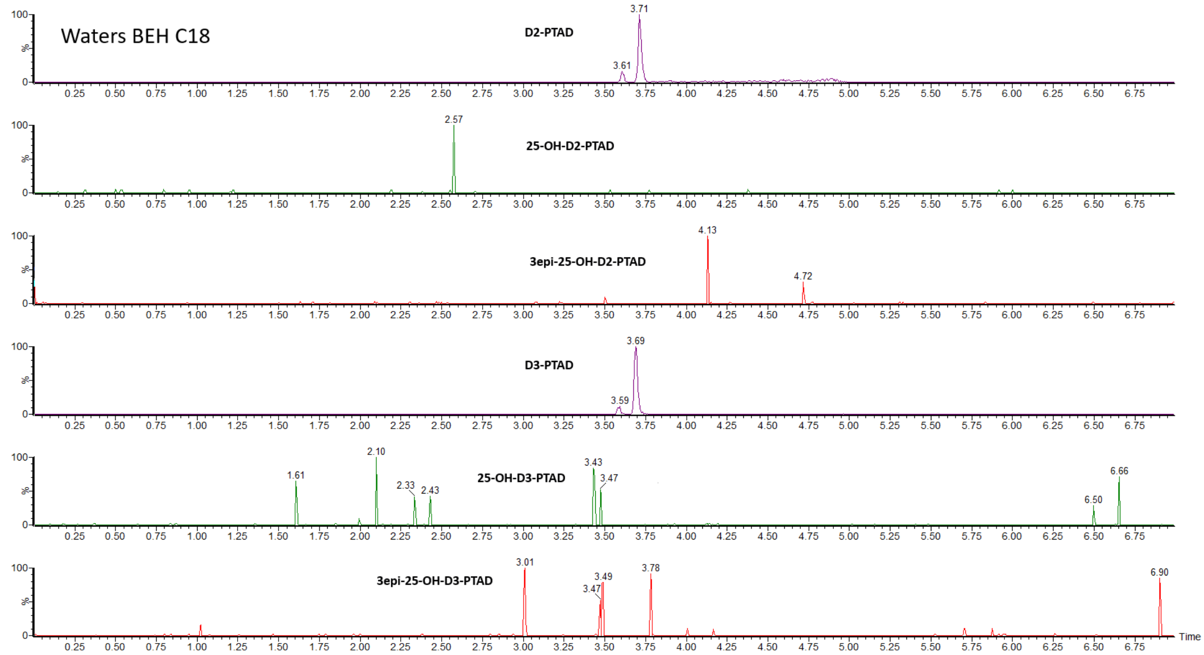

Fig. S2 Chromatogram obtained during column screening on column 2: Waters BEH C18, 3 mm x 100 mm, 1.7 µm. Injection of PTAD-derivatized individual solutions (100 ng/mL, 3 µL). Gradient elution, methanol:water (98:2) containing 10 mM ammonium formate into carbon dioxide. Retention time in minutes

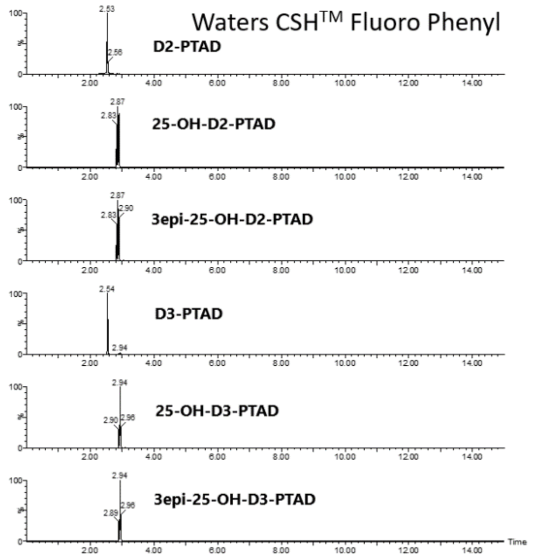

Fig. S3 Chromatogram obtained during column screening on column 3: Waters CSH^{TM} Fluoro Phenyl, 3 mm x 100 mm, 1.7 µm. Injection of PTAD-derivatized individual solutions (100 ng/mL, 3 µL). Gradient elution, methanol:water (98:2) containing 10 mM ammonium formate into carbon dioxide. Retention time in minutes

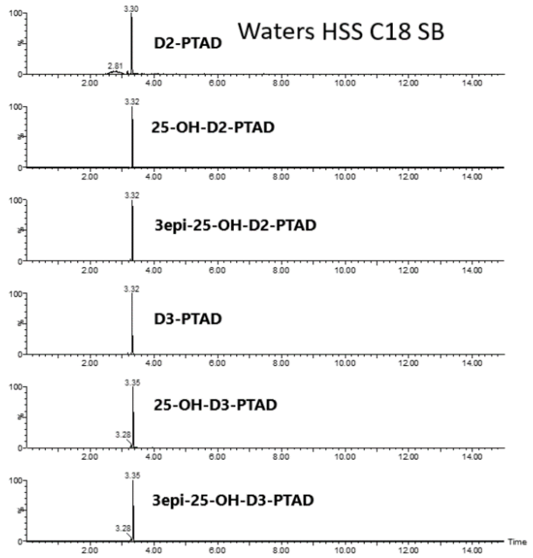

Fig. S4 Chromatogram obtained during column screening on column 4: Waters HSS C18 SB, 3 mm x 100 mm, 1.8 µm. Injection of PTAD-derivatized individual solutions (100 ng/mL, 3 µL). Gradient elution, methanol:water (98:2) containing 10 mM ammonium formate into carbon dioxide. Retention time in minutes

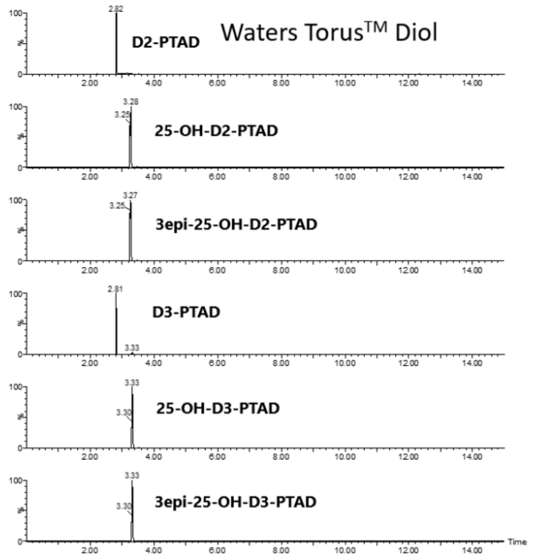

Fig. S5 Chromatogram obtained during column screening on column 5: Waters Torus^{TM} Diol, 3 mm x 100 mm, 1.7 µm. Injection of PTAD-derivatized individual solutions (100 ng/mL, 3 µL). Gradient elution, methanol:water (98:2) containing 10 mM ammonium formate into carbon dioxide. Retention time in minutes

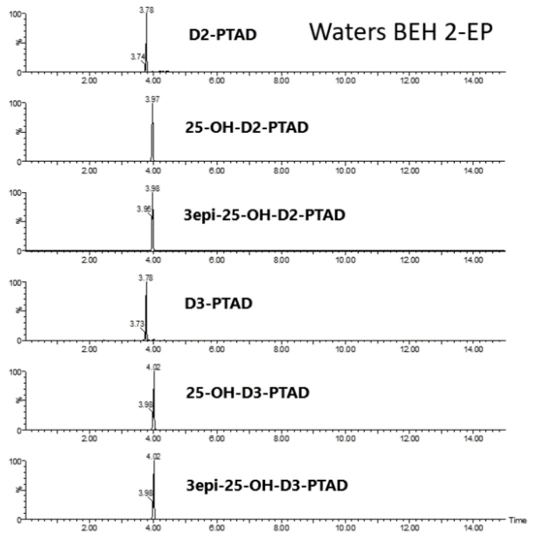

Fig. S6 Chromatogram obtained during column screening on column 6: Waters BEH 2-EP, 3 mm x 100 mm, 1.7 µm. Injection of PTAD-derivatized individual solutions (100 ng/mL, 3 µL). Gradient elution, methanol:water (98:2) containing 10 mM ammonium formate into carbon dioxide. Retention time in minutes

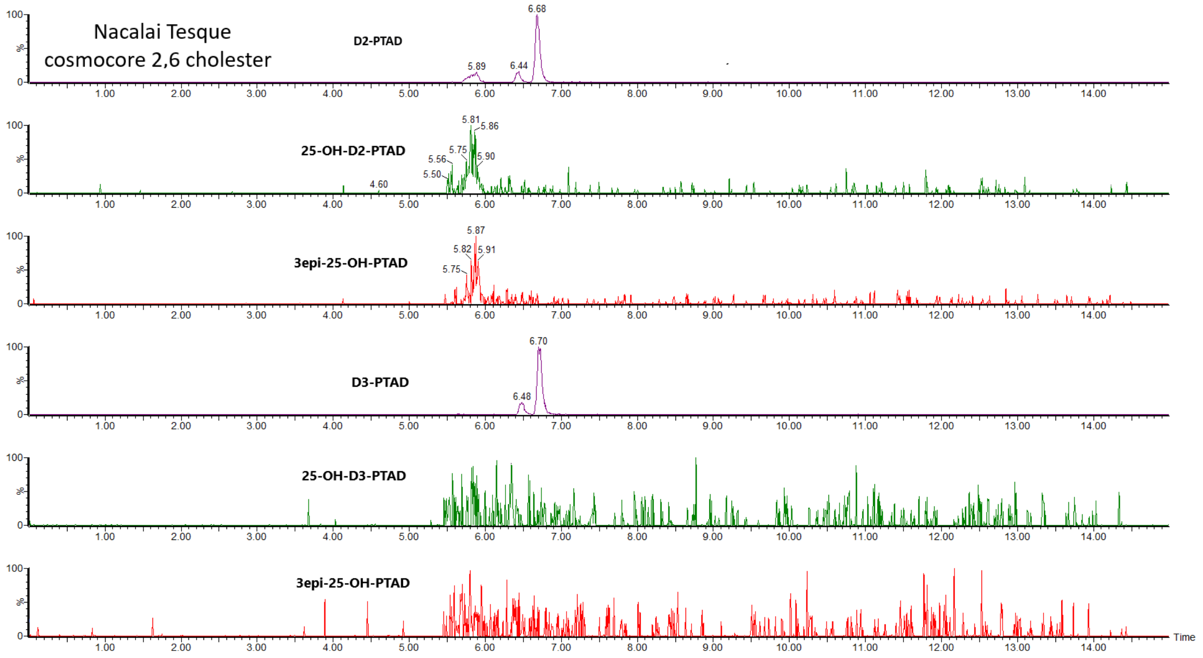

Fig. S7 Chromatogram obtained during column screening on column 7: Nacalai Tesque Cosmocore 2,6 cholester, 2.1 mm x 150 mm, 2.6 µm. Injection of PTAD-derivatized individual solutions (100 ng/mL, 3 µL). Gradient elution, methanol:water (98:2) containing 10 mM ammonium formate into carbon dioxide. Retention time in minutes

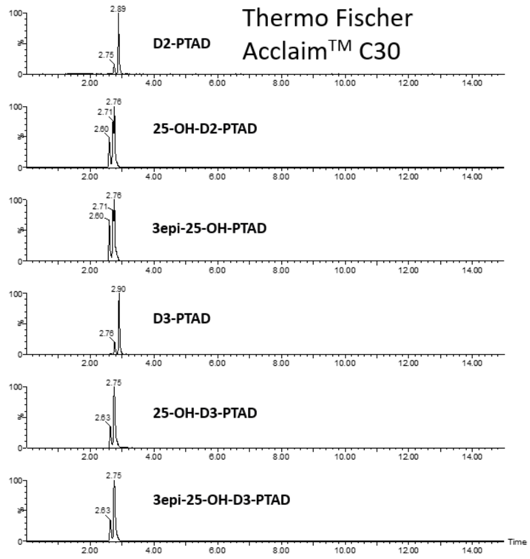

Fig. S8 Chromatogram obtained during column screening on column 8: Thermo Fischer Acclaim™ C30, 2.1 mm x 150 mm, 3 µm. Injection of PTAD-derivatized individual solutions (100 ng/mL, 3 µL). Gradient elution, methanol:water (98:2) containing 10 mM ammonium formate into carbon dioxide. Retention time in minutes

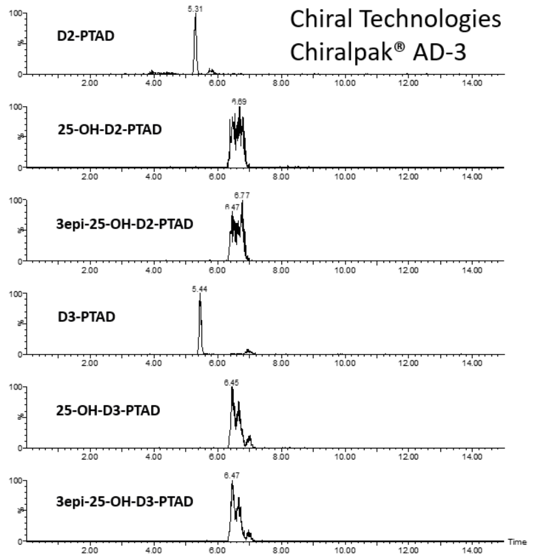

Fig. S9 Chromatogram obtained during column screening on column 9: Chiral Technologies Chiralpak® AD-3, 4.6 mm x 150 mm, 3 µm. Injection of PTAD-derivatized individual solutions (100 ng/mL, 3 µL). Gradient elution, methanol:water (98:2) containing 10 mM ammonium formate into carbon dioxide. Retention time in minutes
